# PD-L1 expression as biomarker of efficacy of PD-1/PD-L1 checkpoint inhibitors in metastatic triple negative breast cancer: A systematic review and meta-analysis

**DOI:** 10.3389/fimmu.2023.1060308

**Published:** 2023-03-06

**Authors:** Muhammad Khan, Kunpeng Du, Meiling Ai, Baiyao Wang, Jie Lin, Anbang Ren, Chengcong Chen, Zhong Huang, Wenze Qiu, Yawei Yuan, Yunhong Tian

**Affiliations:** ^1^ Department of Radiation Oncology, Affiliated Cancer Hospital and Institute of Guangzhou Medical University, Guangzhou, China; ^2^ State Key Laboratory of Respiratory Diseases, Guangzhou Institute of Respiratory Disease, Guangzhou Medical University, Guangzhou, China

**Keywords:** breast cancer, immunotherapy, immune checkpoint inhibitors (ICI), pembrolizumab, atezolizumab, survival, immune-related toxicity

## Abstract

**Background:**

Inhibitors of programmed cell death 1 (PD-1)/programmed cell death ligand 1(PD-L1) checkpoint have been approved for metastatic triple negative breast cancer (mTNBC) in patients positive for PD-L1 expression. Negative results from the recent phase III trials (IMPassion131 and IMPassion132) have raises questions on the efficacy of PD-1/PD-L1 checkpoint inhibitors and the predictive value of PD-L1 expression. Here we attempt to systematically analyze the biomarker value of PD-L1 expression for predicting the response of PD-1/PD-L1 checkpoint inhibitors in mTNBC.

**Materials and methods:**

PubMed database was searched until Dec 2021 for studies evaluating PD-1/PD-L1 checkpoint inhibitors plus/minus chemotherapy in mTNBC. Outcome of interest included objective response rate (ORR), progression-free survival (PFS), and overall survival (OS). Review Manager (RevMan) version 5.4. was used for data-analysis.

**Results:**

In total, 20 clinical trials comprising 3962 mTNBC patients (ICT: 2665 (67%); CT: 1297 (33%) were included in this study. Overall ORR was 22% (95%CI, 14-30%) and significant improvement was observed for PD-L1+ patients (ORR 1.78 [95%CI, 1.45-2.19], p<0.00001) as compared to PD-L1- cohort. Pooled outcome also indicated a significant 1-year PFS and 2-year OS advantage for patients with PD-L1 expression (1-year PFS: ORR 1.39 [95%CI, 1.04-1.85], p=0.02; I^2 =^ 0%; 2-year OS: (ORR 2.47 [95%CI, 1.30-4.69], p=0.006; I^2 =^ 63%). Subgroup analysis indicated that PD-L1 expression can successfully predict tumor response and 2-year OS benefit in mTNBC patients regardless of the type of investigating agent, line of treatment administration, and to some extent the type of treatment. Biomarker ability of PD-L1 expression to predict 1-year PFS was slightly better with pembrolizumab (p=0.09) than atezolizumab (p=0.18), and significantly better when treatment was administered in the first-line setting (OR 1.38 [95%CI, 1.02-1.87], p=0.04) and chemotherapy was added (OR 1.38 [95%CI, 1.02-1.86], p=0.03). Immune-related toxicity of any grade and grade≥3 was 39% (95%CI, 26%-52%) and 10% (95%CI, 8%-13%), respectively.

**Conclusions:**

PD-L1 expression can predict objective response rate and 2-year OS in mTNBC patients receiving PD-1/PD-L1 checkpoint inhibitors. One-year PFS is also predicted in selected patients. PD-L1 expression can be a useful biomarker of efficacy of PD-1/PD-L1 checkpoint inhibitors in mTNBC.

## Introduction

Triple negative breast cancer (TNBC), which constitutes about 15 to 20% of the breast cancer cases, is characterized by the lack of expression for hormone receptors (estrogen receptor [ER-] and progesterone receptor [PR-]) and human epidermal growth factor receptor 2 (HER2) (1–3). Multi-omics studies have further identified that majority of TNBC (about 55%-81%) express highly proliferative basal-like genes which can describe its aggressive nature (3). Prognosis is poor and 3-year recurrence is high despite a greater response to chemotherapy as compared to other breast cancer subgroups (3–5). Metastasis is common and is the major cause of death (2). 5-year survival rate is less than 30% for metastatic TNBC (2).

**Figure 1:**
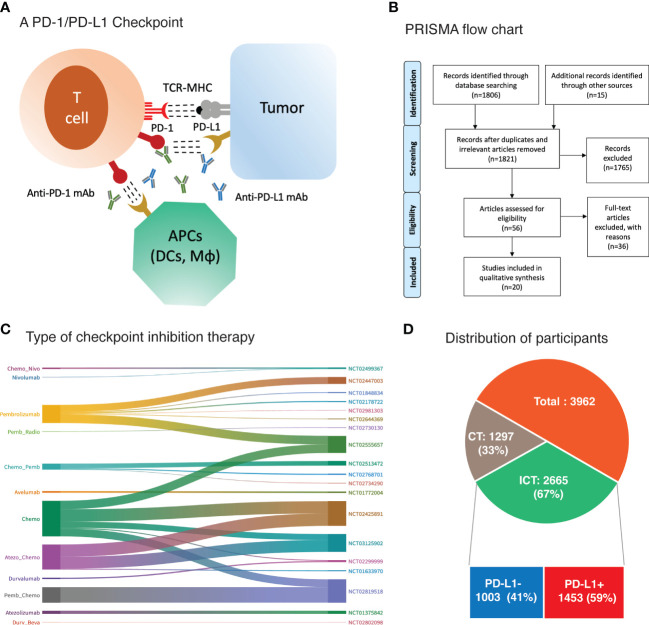
**(A)** Binding of checkpoint proteins, such as PD-1 on T cells and PD-L1 on tumor cells and antigen presenting cells (APCs), keeps T cells from killing tumor cells in the body. Blocking this interaction of PD-1 and PD-L1 with an immune checkpoint inhibitor (anti-PD-L1 or anti-PD-1) allows the T cells to kill tumor cells. DCs, dendritic cells; Mϕ, macrophages **(B)** PRISMA flow diagram of research strategy and study selection. **(C)** Sankey diagram depicting treatment regimen and corresponding trial registration number. **(D)** Main cohorts and number of participants.

Immunotherapy in the form of immune checkpoint inhibition (ICI) was considered as an alternative and complementary cancer treatment option for TNBC due to its high genomic instability, infiltration of tumor-infiltrating lymphocytes (TILs) and elevated expression of programmed cell death protein ligand 1 (PD-L1) (6–8). Programmed cell death protein expressed on the T cells engages its ligand PD-L1 expressed on tumor cells thereby mediating tumor immune escape *via* suppression of antigen-specific T cell immune responses (8). Interruption of this PD-1/PD-L1 interaction with either anti-PD-1 monoclonal antibody (mAb) or anti-PD-L1 mAb results in activation of anti-tumor immune response (8) (**Figure 1A**). Additionally, PD-L1 is also expressed on other tumor-infiltrating immune cells mainly the antigen presenting cells (APCs) such as dendritic cells (DCs) and macrophages among others (9). Studies have identified the indispensable role of PD-L1 expression on such immune cells for the therapeutic efficacy of PD-1/PD-L1 blockade therapy (9, 10). As such, PD-L1 expression on tumor cells as well as host immune cells is evaluated for their biomarker efficacy.

Durable responses were observed in the earlier TNBC trials for anti-PD-1 (pembrolizumab) and anti-PD-L1 (atezolizumab) monoclonal antibodies alone or in combination with chemotherapy (11–15). Results of subsequent phase III trials (IMPassion130 and KEYNOTE-355) had led to approval of these agents in combination with chemotherapy for advanced metastatic TNBC patients (16, 17). The approval was based on the progression free survival benefit observed for TNBC patients receiving the combined treatment particularly in patients positive for PD-L1 expression. However, long term benefit is still unclear and OS results for KEYNOTE-355 is yet to come (17). Moreover, IMPassion131 phase III trial of atezolizumab showed no PFS advantage with combined treatment for PD-L1+ patients (18). These outcomes not only question the long-term benefit of PD-1 inhibitors but also cast doubt on the biomarker value of PD-L1 expression which had formed the basis for FDA approval.

In this study, we have gathered the efficacy data from various TNBC immunotherapy trials to evaluate the status of immunotherapy in advanced TNBC with a focus on PD-L1 expression as biomarker.

## Methods and materials

This systematic-review and meta-analysis was carried out following the updated version 2020 of PRISMA (Preferred Reporting Items for Systematic Reviews and Meta-Analyses) guidelines (19).

### Eligibility criteria

The PICOT (Population, Index prognostic factors, Comparator prognostic factors, Outcomes, Timings, and Settings) system was utilized for identifying the key elements of this review.

◼ **Target population and treatment**: Advanced metastatic triple negative breast cancer (TNBC) patients receiving immunotherapy, mainly PD-1/PD-L1 checkpoint inhibitors, with or without chemotherapy.
◼ **Index Prognostic factors**: PD-L1 expression was the only main index prognostic factor.
◼ **Comparator prognostic factors**: not applicable for this review.
◼ **Outcome of interest:** Main efficacy outcomes of interest included objective response rate (ORR), progression-free survival (PFS) and overall survival (OS). Definition of ORR and PFS were based on Response Evaluation Criteria in Solid Tumors (RECIST) version 1.1. Frequencies of adverse events graded according to the National Cancer Institute Common Terminology Criteria for Adverse Events (CTCAE), version 4.0. was also evaluated between the treatment cohorts.
◼ **Timing**: PD-L1 expression was assessed before immunotherapy
◼ **Setting:** Cancer hospitals and treatment centers.

### Study design and language restrictions

Single arm studies that provided treatment comparison based on PD-L1 status and comparative clinical trials (CTs) that compared PD-1/PD-L1 checkpoint inhibitors +/- chemotherapy to chemotherapy alone were pursued with English language restrictions.

### Research strategy and study selection

PubMed was formally searched with several key terms until Dec, 2021. Further potential studies were identified through screening references of relevant articles. A step-wise procedure comprising retrieving, organizing, and screening was followed by two reviewers to select studies meeting the eligibility criteria. Disagreements were solved after consulting the third author.

### Data extraction

Modified form “The Cochrane Collaboration Data Collection form for RCTs” was used for data extraction that is available from Cochrane website. Characteristics of the included studies and attributes of participants were extracted that included first author, publication year, trail designation, national clinical trial (NCT) registration number, trial design, number and type of participants, and treatment type. Participants’ attributes included age, PD-L1 expression status, PD-L1 assessment assay, and the lines of previous therapy for metastatic disease. Relevant outcome data was also extracted for performing meta-analysis.

### Quality assessment

The Cochrane Collaboration Tool were used to assess the quality of included randomized controlled trials (20). Assessments included sequence generation, allocation of sequence concealment, blinding of participants and personnel, blinding of outcomes and assessments, incomplete outcome data, selective outcome reporting, and other biases. Non-randomized clinical trials were assessed with the methodological index for non-randomized studies (MINORS), which contains eight items of assessment and is recognized as the most appropriate guideline to evaluate the methodological quality of non-randomized trials (21). As a meta-analysis of biomarker assessment, the risk of bias was also assessed by “The Quality In Prognosis Studies (QUIPS) tool” for studies reporting biomarker analysis (22, 23). The QUIPS tool used six important domains (1): study participation (2), study attrition (3), prognostic factor measurement (4), outcome measurement (5) study confounding, and (6) statistical analysis and reporting. Quality of evidence assessment was carried out with The Grading of Recommendations Assessment, Development and Evaluation (GRADE) (24). Major outcomes were graded as high, moderate, low, or very low depending upon the following elements: study design, risk of bias, inconsistencies, imprecision of the results, indirectness, and publication bias.

### Measurement of treatment effect and data synthesis

Number of events for objective response rates (ORR: Complete Responses + Partial Responses) and adverse events were pooled using Mantel-Haenszel method (19, 25). Pooling of number of events for single-arm studies was carried out with the help of a step-by-step guide for meta-analysis of descriptive data analysis developed by Neyeloff, et al. (26). Hazard ratios for PFS and OS were recorded directly from the study or extracted from the K-M curves using the Digital Equalizer and methods for incorporating summary time-to-event data into the meta-analysis according to Tierney et al. (27, 28). Pooling of HRs was done by applying inverse variance method (19, 25). Heterogeneity was assessed using Chi^2^ test and I^2^ value and graded as low (I^2 =^ 25%), moderate (I^2 =^ 50%), and high (I^2 =^ 75%) according to I^2^ values (29). Random effects model was adopted when heterogeneity exceeded 50% (I^2 ≥^50%). Significance level was set at p<0.05.

## Results

The research strategy and study selection are illustrated in **Figure 1B**. A total of 1806 published studies were identified upon initial database search. Title and abstract screening excluded 1765 studies for various reasons including duplicates, irrelevant, lacking data of main outcomes, and not target agent/s, etcetera. Further scrutiny for eligibility and full text reading yielded 20 studies that were included in this meta-analysis (11–18, 30–41). General characteristics of the included studies and participants are presented in **Table 1**. The detailed treatment regimen is highlighted in **Figure 1C**, along with the national clinical trial number for each study. Overall, 3962 patients with mTNBC were available for analysis as depicted in **Figure 1D**. A total of 2665 (67%) participants had received PD-1 (nivolumab and pembrolizumab) or PD-L1 inhibitor (avelumab, atezolizumab, and durvalumab) as monotherapy or in combination with chemotherapy. This cohort was described as ICT cohort. While the remaining (33%) were administered with chemotherapy alone (CT cohort). Patients with PD-L1+ expression were 1453 (59%) and 1003 (41%) patients were negative for PD-L1 expression in the ICT cohort. Pembrolizumab was investigated in 12 studies; atezolizumab in 4; durvalumab in 2; and avelumab in only one study (**Figure 1C** and **Table 1**). Pembrolizumab was also administered with induction chemotherapy in few trials (31, 32) and was followed by radiotherapy in a single trial (33). Likewise, three studies had evaluated immune modulating agents along with pembrolizumab (34–36). However, these studies had only reported response rates in mTNBC and were not incorporated into the biomarker analysis due to lack of relevant data (33–36). PD-L1 expression was assessed with PD-L1 IHC 22C3 pharmDx assay in studies involving pembrolizumab measuring the PD-L1 expression on tumor cells as well as immune cells which was termed as combined positive score (CPS) (11, 12, 17, 30, 41). Atezolizumab studies (Phase III RCTs) utilized VENTANA PD-L1 (SP142) assay assessing the PD-L1 expression on immune cells (IC) alone as the phase I trial had revealed a significant improvement based on the PD-L1 expression on immune cells and not tumor cells (TC) (15, 16, 18). Durvalumab also showed better predictive value of PD-L1 expression on immune cells in a phase II trial after an initial pilot study demonstrated no significant predictive value of PD-L1 expression assessed on tumor cells (38, 39). Details are highlighted in **Table 1**.

**Table 1:** General characteristics of the included randomized controlled trials.

First Author OR Trial designation	NCT number	Year	Trial Design	No. of patients Total (ICT/CT)	Age (median, range)	Anti PD-1/PD-L1 agent	PD-L1 assessment assay	PD-L1 expression score	Line of Therapy
Adams, et al.	NCT01633970	2019	Phase Ib Clinical Trial	33	55 (32–84)	Atezolizumab	VENTANA PD-L1 (SP142) assay	TC/IC	1L=13/≥2L
Anders, et al.	NCT02768701	2019	Phase II Clinical Trial	40	54.5 (33–82)*	Pembrolizumab	NA	NA	≥2L
O'Day, et al.	NCT02981303	2019	Phase II Clinical Trial	12		Pembrolizumab	NA	NA	≥2L
Page, et al.	NCT02734290	2019	Phase I/II Clinical Trial	14		Pembrolizumab	NA	NA	1L/≥2L
McArthur, et al.	NCT02730130	2018	Phase II Clinical Trial	17	52 (37–73)	Pembrolizumab	NA	NA	1L/≥2L
Spira, et al.	NCT02178722	2017	Phase I/II Clinical Trial	39		Pembrolizumab	NA	NA	1L/≥2L
INSPIRE	NCT02644369	2018	Phase II Clinical Trial	19	40	Pembrolizumab	NA	NA	1L/≥2L
KEYNOTE-086A	NCT02447003	2019	Phase II Clinical Trial	170	53.5 (28–85)	Pembrolizumab	PD-L1 IHC 22C3 pharmDx assay	CPS	≥2L
KEYNOTE-086B	NCT02447003	2019	Phase II Clinical Trial	84	52.5 (26–91)	Pembrolizumab	PD-L1 IHC 22C3 pharmDx assay	CPS	IL
JAVELIN	NCT01772004	2018	Phase Ib Clinical Trial	58	52.5 (31–80)	Avelumab	PD-L1 IHC 73-10 pharmDx	TC/IC	≥2L
Emens, et al.	NCT01375842	2019	Randomized, Open-label, Phase I Clinical Trial	115	53 (29–82)	Atezolizumab	VENTANA PD-L1 (SP142) assay	TC/IC	1L=21/≥2L
Quintela-Fandino, et al.	NCT02802098	2020	Pilot Clinical Trial	9	54.1 (34.5-77.4)	Durvalumab	VENTANA PD-L1 (SP263) assay	TCs	≥2L
ENHANCE 1	NCT02513472	2021	Phase Ib/II Clinical Trial	167	56 (32–88)	Pembrolizumab	PD-L1 IHC 22C3 pharmDx assay	CPS	1L=66/≥2L
TONIC	NCT02499367	2019	Phase II Clinical Trial	54	51 (29–41)	Nivolumab	PD-L1 IHC 22C3 pharmDx assay	NA	≥2L
KEYNOTE-012	NCT01848834	2016	Phase Ib Clinical Trial	27	50.5 (29–42)	Pembrolizumab	PD-L1 IHC 22C3 pharmDx assay	Stroma & Tumor cells	≥2L
KEYNOTE-119	**N**CT02555657	2021	Randomized, Open-label, Phase III trial	622 (312/310)	50 (43–59)/ 53 (44–61)	Pembrolizumab	PD-L1 IHC 22C3 pharmDx assay	CPS	≥2L
SAFIR02-BREAST IMMUNO	NCT02299999	2021	Randomized, Open-label, Phase II Clinical Trial	82 (47/35)		Durvalumab	VENTANA PD-L1 (SP142) assay	IC	1L
IMPassion130	NCT02425891	2020	Multicentre, Multinational, Randomised (1:1), Double-blind, Placebo-controlled, Phase III Trial	902 (451/451)	55 (46–64)/ 56 (46-65)	Atezolizumab	VENTANA PD-L1 (SP142) assay	IC	1L
KEYNOTE-355	NCT02819518	2020	Multicentre, Multinational, Randomised (2:1), Double-blind, Placebo-controlled, Phase III Trial	847 (566/281)	53 (44–63)/ 53 (43–63)	Pembrolizumab	PD-L1 IHC 22C3 pharmDx assay	CPS	1L
IMPassion131	NCT03125902	2020	Randomised (2:1), Double-blind, Placebo- controlled, Phase III Trial	651 (431/220)	54 (22–85)/53 (25–81)	Atezolizumab	VENTANA PD-L1 (SP142) assay	IC	1L

NCT, national clinical triial; TNBC, triple negative breast cancer; PD-1/PD-L1, programmed cell death protein 1/ programmed cell death ligand 1; CPS, combined positive score; IC, immune cells; TCs, Tumor cells; ICT, immune-chemotherapy; CT, chemotherapy.

### Quality assessment of the studies and evidence

Quality assessment was carried out using The Cochrane Collaboration Tool. Only three RCTs were double-blinded exhibiting a low risk of bias (16–18). Detail assessment is highlighted in **Supplementary Table 1**. Quality assessment of the included studies based on the MINORS and QUIPS tools are detailed in **Supplementary Tables 2, 3**. MINORS demonstrated that about 80% of the studies (11–18, 30, 32, 33, 36–41) had low risk of bias (score 10-15) and 20% exhibited a moderate risk of bias (score <10) (31, 34, 35). QUIPS tool, which assess the risk of bias in the studies involving assessment of prognostic factors, showed 66% of the studies had low risk of bias. Nonetheless, 33% of the studies demonstrated high risk of bias in terms of participants (30), study attrition (30), prognostic factor (15, 30), and confounding (40, 41). Quality of evidence assessment based on the GRADE quality tool is presented in **Supplementary Table 4**, which indicated that main three outcomes carried low risk of bias and were considered of critical importance with moderate to low certainty.

### Objective response rate

Objective response rates for the entire mTNBC cohort were available from 19 studies involving 2617 patients. Pooled analysis revealed an ORR of 22% (95%CI, 14-30%) (**Supplementary Figure 1**). When compared to CT alone, the ORR was higher in ICT cohort (OR 1.32 [95%CI, 1.13-1.55], p=0.0007) (**Supplementary Figure 2A**).

**Figure 2:**
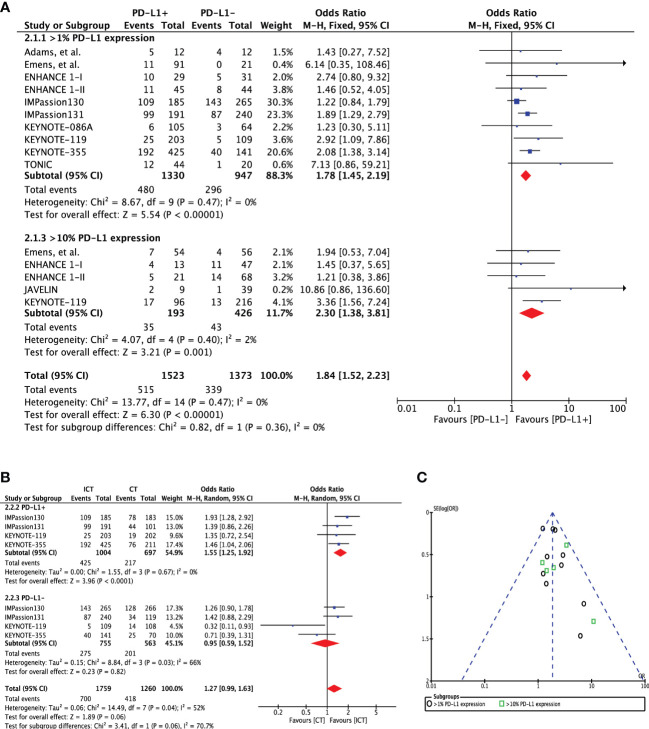
**(A)** Comparison of objective response rate (ORR) in advanced metastatic TNBC (mTNBC) patients treated with PD-1 checkpoint inhibition therapy based on PD-L1 expression. **(B)** Comparison of objective response rate (ORR) in advanced metastatic TNBC (mTNBC) patients (PD-L1+ & PD-L1-) between patients receiving PD-1 checkpoint inhibitors plus chemotherapy (ICT) and CT alone. **(C)** Funnel plot of publication bias assessment in objective response rate (ORR) analysis.

Overall, nine studies (n=2277) reported ORR for PD-L1 expression difference. A direct comparison of PD-L1+ cohort (n=1330) to PD-L1- cohort (n=947) showed significantly improved ORR in patients with PD-L1 expression (OR 1.78 [95%CI, 1.45-2.19], p<0.00001) (**Figure 2A**). There was no heterogeneity (Chi² = 8.67, df = 9 (P = 0.47); I² = 0%) and publication bias observed for this outcome as shown in **Figure 2C**. The ORR was even higher in patients with ≥10% PD-L1 expression (OR 2.30 [95%CI, 1.38-3.81], p=0.001). Moreover, there was evidence of an increasing probability of objective response with increasing PD-L1 expression in the KEYNOTE-012 trial (p=0.028) (14). Four studies (n=1701) have reported ORR for treatment difference (ICT versus CT) in PD-L1+ (n=3019) and PD-L1- cohorts (n=1318). Pooled analysis showed significantly improved ORR for PD-L1+ positive patients in the ICT cohort (ORR 1.55 [95%CI, 1.25-1.92], p<0.0001) without any heterogeneity (I² = 0%) (**Figure 2B**). However, PD-L1- TNBC cohort showed no significant change in ORR for the treatment difference (ORR 0.95 [95%CI, 0.59-1.52], p=0.82). Difference between these two subgroups was close to significance (Chi² = 3.41, df = 1 (P = 0.06), I² = 70.7%).

### Progression-free survival

**Figure 3:**
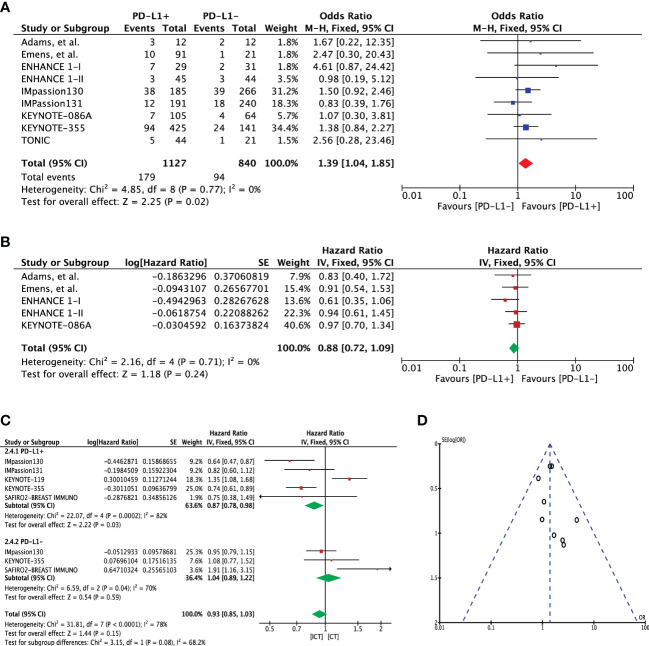
**(A)** Comparison of 1-year progression-free survival (PFS) in advanced metastatic TNBC (mTNBC) patients treated with PD-1 checkpoint inhibition therapy based on PD-L1 expression. **(B)** Comparison of progression-free survival (PFS) in advanced metastatic TNBC (mTNBC) patients treated with PD-1 checkpoint inhibition therapy based on PD-L1 expression. **(C)** Comparison of progression-free survival (PFS) in PD-L1+ and PD-L1- metastatic TNBC (mTNBC) patients between patients receiving PD-1 checkpoint inhibitors plus chemotherapy (ICT) and CT alone. **(D)** Funnel plot of publication bias assessment in 1-year progression-free survival (PFS) analysis.

Progression-free survival was assessed using 1-year and overall PFS outcomes. One-year PFS event rates for PD-L1 expression cohorts were available in eight studies (n=1967). Pooled outcome indicated a significant 1-year PFS advantage for patients with PD-L1 expression (OR 1.39 [95%CI, 1.04-1.85], p=0.02; I^2 ^= 0%) (**Figure 3A**). There was no heterogeneity (Chi² = 4.85, df = 8 (P = 0.77); I² = 0%) and publication bias observed for this outcome as shown in **Figure 3D**. A direct comparison of overall PFS in these cohorts was available in 5 studies. Extracted hazard ratios were pooled which showed no difference between the cohorts (HR 0.88 [95%CI, 0.72-1.09], p=0.24; I^2 ^= 0%) (**Figure 3B**). In KEYNOTE-012 trial, significant reduction in the hazard to progression or death was shown with increasing PD-L1 expression (p=0.012) (14).

Five studies (n=3104) reported PFS for treatment difference (ICT=1807 versus CT=1297). Pooled analysis showed significantly improved PFS for PD-L1+ positive patients in the ICT (PFS 0.87 [95%CI, 0.78-0.98], p=0.03); however, heterogeneity was high (I² = 82%) (**Figure 3C**). Application of random effects model resulted in loss of significance difference (HR 0.84 [95%CI, 0.62-1.15], p=0.28) (**Supplementary Figure 3A**). On the other hand, PD-L1- TNBC cohort showed no significant change in PFS for the treatment difference (HR 1.04 [95%CI, 0.89-1.22], p=0.59). Difference between these two subgroups was close to significance (Chi² = 3.15, df = 1 (P = 0.08), I² = 68.2%) (**Figure 3C**). In an attempt to address the heterogeneity, sensitivity analysis was carried out with excluding the KEYNOTE-119 trial which included patients whom had received more than two lines of treatments for metastatic disease. Upon exclusion, pooled analysis showed significant improvement in PFS with no heterogeneity (HR 0.73 [95%CI, 0.64-0.84], p<0.0001, I² = 0%), which also resulted in significant subgroup difference (Chi² = 10.82, df = 1 (P = 0.001), I² = 90.8%) (**Supplementary Figure 3B**). A similar outcome was demonstrated when the entire mTNBC cohort (regardless of PD-L1 expression status) was considered for the treatment difference (**Supplementary Figures 2B, C**).

### Overall survival

**Figure 4:**
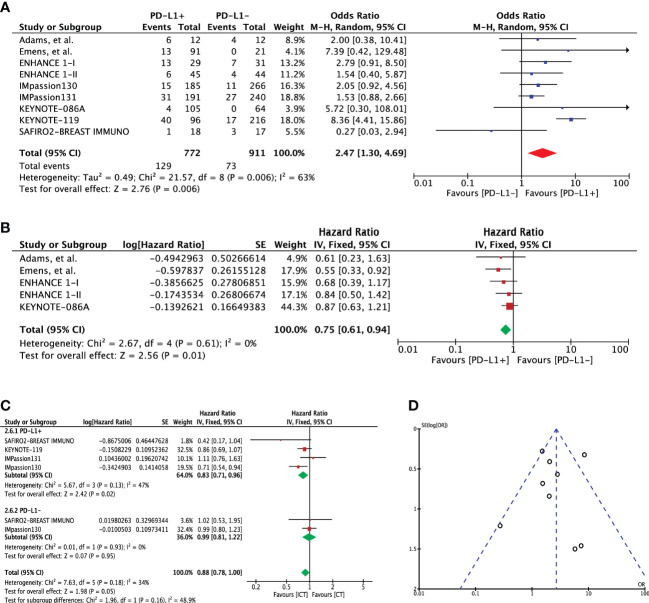
**(A)** Comparison of 2-year overall survival (OS) in advanced metastatic TNBC (mTNBC) patients treated with PD-1 checkpoint inhibition therapy based on PD-L1 expression. **(B)** Comparison of overall survival (OS) in advanced metastatic TNBC (mTNBC) patients treated with PD-1 checkpoint inhibition therapy based on PD-L1 expression. **(C)** Comparison of overall survival (OS) in PD-L1+ and PD-L1- metastatic TNBC (mTNBC) patients between patients receiving PD-1 checkpoint inhibitors plus chemotherapy (ICT) and CT alone. **(D)** Funnel plot of publication bias assessment in 2-year overall survival (OS) analysis.

Overall survival was assessed using 2-year and overall survival outcomes. Two-year OS event rates for PD-L1 expression cohorts were available in eight studies (n=1683). Pooled outcome indicated a significant 2-year OS advantage for patients with PD-L1 expression (OR 2.47 [95%CI, 1.30-4.69], p=0.006; I^2 ^= 63%) (**Figure 4A**). Heterogeneity was high; hence, random effects model was adopted. No publication bias was observed for this outcome as shown in **Figure 4D**. A direct comparison of overall survival for these two cohorts was available in 5 studies. Extracted hazard ratios were pooled which also indicated a significant difference between the cohorts (HR 0.75 [95%CI, 0.61-0.94], p=0.01; I^2 ^= 0%) (**Figure 4B**). Four studies (n=2257) reported OS for treatment difference (ICT=1241 versus CT=1061). Pooled analysis showed significantly improved OS for PD-L1+ positive patients in the ICT (HR 0.83 [95%CI, 0.71-0.6], p=0.02); however, heterogeneity was high (I² = 82%) (**Figure 4C**). On the other hand, PD-L1- TNBC cohort showed no significant change in OS for the treatment difference (HR 0.99 [95%CI, 0.81-1.22], p=0.95). Difference between these two subgroups showed no significance (Chi² = 1.96, df = 1 (P = 0.16), I² = 48.9%). Comparison of overall survival when the entire mTNBC cohort (regardless of PD-L1 expression status) was considered showed no survival advantage for ICT (**Supplementary Figure 2D**).

### Subgroup analysis

**Figure 5:**
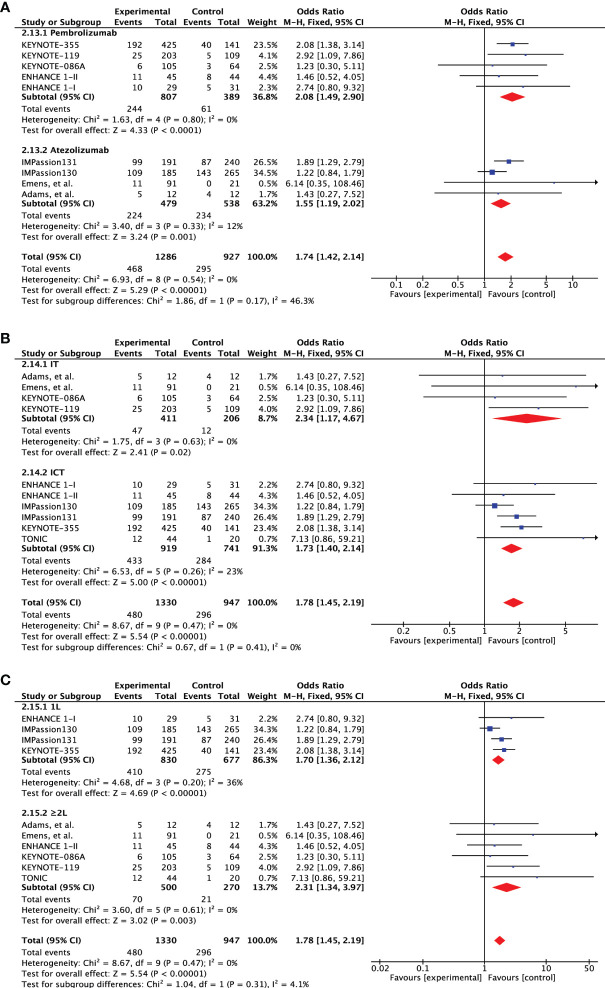
Subgroup analysis based on objective response rate (ORR). **(A)** Forest plot of comparison of objective response rate (ORR) between PD-L1+ and PD-L1- metastatic triple-negative breast cancer treated with pembrolizumab and atezolizumab, **(B)** PD-1 checkpoint inhibitors alone (IT) and PD-1 checkpoint inhibitors plus chemotherapy (ICT), and **(C)** treatment administered in first- and second-line setting.

As the included studies were heterogenous according to the type of therapeutic agents, line of treatment and the addition of chemotherapy, we further carried out subgroup analysis to investigate the predictive significance of PD-L1 expression (**Table 2**). First of all, we considered the studies that either included pembrolizumab or atezolizumab as the investigating agent. The response was slightly higher in pembrolizumab cohort (OR 2.08 [95%CI, 1.49-2.90], p<0.0001) than atezolizumab (OR 1.55 [95%CI, 1.19-2.02], p=0.001) with no significant difference between the two agents (subgroup differences: Chi² = 1.86, df = 1 (P = 0.17), I² = 46.3%) (**Figure 5A**). Pembrolizumab showed to improve the 1-year PFS (OR 1.44 [95%CI, 0.94-2.21], p=0.09) as compared to atezolizumab (p=0.18) but still no significant differences were observed for subgroups (p=0.74) (**Supplementary Figure 4A**). While 2-year OS was significantly increased with PD-L1 expression in both subgroups (Pembrolizumab: OR 3.97 [95%CI, 1.61-9.80], p=0.003; Atezolizumab: OR 1.76 [95%CI, 1.14-2.72], p=0.01; subgroup difference: p=0.11) (**Supplementary Figure 5A**). This comparison also the rules out the impact of different PD-L1 expression assays (SP142 & 222C3) and scoring algorithms (CPS vs IC) on the overall significance of PD-L1 expression.

**Table 2:** Subgroup analysis.

Category	Subgroups	Significant improvement with PD-L1 expression					
		ORR	diff*	1-year PFS	diff	2-year OS	diff
Type of agents	Pembrolizumab	**Y**	p=0.17	**X** (p=0.09)	p=0.74	**Y**	p=0.11
	Atezolizumab	**Y**		**X**		**Y**	
Addition of chemotherapy	IT	**Y**	p=0.41	**X**	p=0.92	**X** (p=0.07)	p=0.38
	ICT	**Y**		**Y**		**Y**	
Line of therapy	1L	**Y**	p=0.31	**Y**	p=0.90	**Y**	p=0.08
	≥2L	**Y**		**X**		**Y**	

ORR, objective response rate; PFS, progression-free survival; OS, overall survival; IT, immunotherapy; ICT, immunotherapy plus chemotherapy; L, line of therapy.

diff * indicates the statistical difference between two subgroups.

Y indicates significant association with PD-L1 expression.

X indicates no significant association with PD-L1 expression.

Second, we also evaluated the effect of chemotherapy addition to PD-1 inhibitors by considering studies evaluating single agent (IT) and combined therapy (ICT). The response was almost double for both treatment strategies (IT: OR 2.34 [95%CI, 1.17-4.67], p=0.02; ICT: OR 1.73 [95%CI, 1.40-2.14], p<0.00001) with no statistical difference (subgroup differences: Chi² = 0.67, df = 1 (P = 0.41), I² = 0%) (**Figure 5B**). IT failed to improve 1-year PFS and was significantly enhanced by ICT (OR 1.38 [95%CI, 1.02-1.86], p=0.03) but still no significant differences were identified (p=0.92) (**Supplementary Figure 4B**). A similar outlook was demonstrated for 2-year OS but the difference in IT cohort was close to significance (p=0.07) (**Supplementary Figure 5B**).

Lastly, we also evaluated if the significance of PD-L1 expression is maintained when therapy is administered in the second line setting? Predictive ability of PD-L1 expression was slightly better in first line setting (OR 1.70 [95%CI, 1.36-2.12], p<0.00001) as compared to the second line administration (OR 2.31 [95%CI, 1.34-3.97], p=0.003) (**Figure 5C**). However, the effect showed no statistical difference. On the other hand, improvement in 1-year PFS was only seen in the first-line setting (OR 1.38 [95%CI, 1.02-1.87], p=0.04) (**Supplementary Figure 4C**). Although both subgroups showed significant 2-year OS advantage, the subgroups difference tended towards significance (p=0.08) (**Supplementary Figure 5C**).

### Safety concerns

Toxicity data were reported in the form of treatment-related adverse events (TRAEs) and immunotherapy-related adverse events (IRAEs).

### General toxicity

Based on the results from 7 studies (n=1401), frequency of any grade treatment-related adverse events was 71% (95%CI, 49%-92%) as shown in **Supplementary Figure 6A**. Frequency of any grade≥3 was 30% (95%CI, 17%-44%) (**Supplementary Figure 6B**). Additionally, general toxicity of ICT to CT alone was also evaluated. No difference in TRAEs (OR 1.58 [0.99, 2.52], p=0.06) or any grade ≥3 TRAEs (OR 0.90 [0.47, 1.73], p=0.75) was demonstrated (**Supplementary Figure 7**).

### Toxicity of special interest

Toxicity of special or clinical interest mainly included adverse events associated with immunotherapy. Based on the results from 10 studies (n=2290), frequency of any grade treatment-related adverse events was 39% (95%CI, 26%-52%) (**Supplementary Figure 8A**). Frequency of any grade≥3 was 10% (95%CI, 8%-13%) (**Supplementary Figure 8B**). Compared to CT alone, toxicity of special interest was significantly more common in the IT group (OR 2.89 [1.56, 5.38], p=0.0008) (**Supplementary Figure 7**). A significant rise was also observed in grade ≥ 3 irAEs as well (OR 2.53 [1.66, 5.53], p=0.02).

## Discussion

Immune checkpoint inhibition (ICI) therapy has been extensively evaluated in the management of triple negative breast cancer patients. ICI as monotherapy or in combination with chemotherapy have demonstrated anti-cancer activity in early-stage as well as metastatic TNBC (11–18, 42–46). Our study result indicates a 22% response rate for PD-1/PD-L1 checkpoint inhibitors as monotherapy or in combination with chemotherapy in mTNBC. The later approach was also shown superior to chemotherapy alone in terms of ORR and PFS. However, no overall survival advantage was demonstrated for the combined approach.

PD-L1 expression has been regarded as prognostic factor in various cancers (47–50). Its biomarker value to predict response to PD-1/PD-L1 checkpoint inhibition has also been acknowledged across cancers (50–55). Among breast cancer subtypes, TNBC exhibit higher tumor mutational burden (TMB), tumor infiltrates of lymphocytes (TILs) and PD-L1 expression, indicating its candidacy for immune checkpoint inhibition therapy (6–8). In TNBC, PD-L1 expression as biomarker have failed to predict pathological response rate in the neoadjuvant setting and long-term OS benefit in the metastatic setting (56). Our study systematically accumulated data to analyze the biomarker significance of PD-L1 expression in the metastatic TNBC patients. The outcome revealed that PD-L1 expression can significantly predict objective response, 1-year PFS, 2-year OS and overall survival. However, overall PFS was not significantly different between these two groups based on the direct comparison analysis. Nonetheless, the studies that provided the direct PFS comparison between PD-L1+ and PD-L1- patients were comprised of smaller number of participants (12, 13, 15, 30). While major phase III trials reported treatment difference in each subgroup (PD-L1+/- cohort) separately with no direct comparison between the PD-L1+ and PD-L1- patients (16–18). Besides, a much larger population cohort would be required to detect a PFS difference of any significant value in a population with such a heterogenous and extensive disease burden as mTNBC (57). On the other hand, OS is affected by several other patient factors other than the treatment as compared to PFS which is a direct measure of clinical performance (58). In the treatment comparative analysis, the response rate, PFS and OS was significantly higher with ICT in PD-L1 positive population. While PD-L1 negative patients failed to derive such benefit. Overall, these outcomes strongly underline biomarker value of PD-L1 expression in mTNBC.

PD-L1 expression was assessed on the tumor cells and/or the tumor infiltrating lymphocytes and macrophages (11–18, 30, 32, 38, 39, 41). Studies involving pembrolizumab had reported the combined positive score (CPS), defined as the ratio of PD-L1–positive cells (tumor cells, lymphocytes, and macrophages) out of the total number of tumor cells × 100 (11, 12, 17, 30, 41). The prognostic and predictive efficacy of atezolizumab was initially assessed with PD-L1 expression on the tumor cells (TC) and immune cells (IC) (13, 15). However, the phase Ib trial conducted by Emens, et al. indicated a significant predictive value for PD-L1 expression on immune cells only (15). Therefore, subsequent phase III trials had only evaluated the predictive efficacy of atezolizumab based on the IC-derived PD-L1 which was defined as a percentage of tumor area (consisting of TC and associated intra-tumoral and continuous peri-tumoral stroma) occupied by IC with discernible PD-L1 staining of any intensity (16, 18). Subgroup analysis revealed that predictive value of PD-L1 expression was maintained regardless of the scoring algorithm used. PD-L1 expression was able to predict ORR and 2-year OS to both of these agents. Although a near to significant improvement in 1-year PFS was observed for pembrolizumab in PD-L1+ patients, PD-L1 expression failed to predict 1-year PFS for these two subgroups. This subgroup analysis also established that the type of detection assay (SP142 used for atezolizumab & 222C3 for pembrolizumab) also had no impact on the clinical activity. This outcome is in concordance with the observations made in comparative analysis of these immunohistochemistry assays in IMPassion130 study, which indicated significant prevalence differences (prevalence rates for PD-L1 IC ≥1% cutoff for SP142, SP263, and 22C3 were 46.4%, 74.9%, and 73.1%, respectively. And for PD-L1 22C3 CPS ≥1 was 80.9%) but similar clinical activity with A+nP vs P+nP for all four subgroups (SP142 IC ≥1%, SP263 IC ≥1%, 22C3 IC ≥1%, and 22C3 CPS ≥1; PFS HR = 0.60 to 0.68; OS HR = 0.74 to 0.79) (59). These observations indicate that the lack of analytical equivalency among PD-L1 expression detection assays exert no impact on the biomarker ability of PD-L1 expression in predicting clinical activity. PD-L1 expression also varies between primary tumor and metastatic sites as previously been reported for mTNBC (60, 61). The specific site of PD-L1 assessment was not clearly reported in these trials but a *post hoc* analysis of IMPassion130 revealed a difference in PD-L1 expression between primary tumor and metastatic sites (44% vs 36%, p=0.014) of mTNBC patients with no apparent influence on the predictive value of PD-L1 expression (59). A negative impact has been reported for number of previous lines of therapy on the response to PD-1 checkpoint inhibitors in lung cancer and melanoma (62–64). Likewise, overall response was higher in mTNBC patients receiving PD-1/PD-L1 checkpoint inhibitors as first line treatment compared to the patients previously treated for metastatic disease (12–16). However, previous therapy had no effect on the biomarker efficacy of PD-L1 expression according to the outcomes of our study. Nonetheless, 1-year PFS could only be predicted by PD-L1 expression when the treatment was administered in the first line setting.

Moreover, use of PD-1/PD-L1 checkpoint inhibitors in mTNBC have shown durable responses as monotherapy but with high heterogeneity from study to study and its supremacy over chemotherapy alone is controversial (11–18). In fact, combined approach has also yield contradictory results in phase III trials in terms of PFS and OS (16, 18). Although PD-L1 expression was able to predict clinical response when analysis was restricted to single-agent and combined approach separately, 1-year PFS and 2-year OS was only predicted when immunotherapy was combined with chemotherapy. It may indicate that the two treatments are applied in combination may induce strong and long-term responses which also result in survival benefits. In addition, preclinical evidence also indicates a synergistic interplay between these two treatments. Chemotherapeutic agents have shown augment mutational load and neoantigen presentation, suppress the immune-suppressive cells, sensitize tumor cells to effector cytokines produced by T cells, induce the expression of major histocompatibility complex class I (MHC-I) and PD-L1 on tumor cells (65–73). These outcomes firmly support the use of PD-L1 expression as prognostic and predictive of response to immune checkpoint inhibition in mTNBC. As expected, the combination of two treatments has led to added toxicity mainly observed in the form of immune-related adverse events. Toxicity associated with immune checkpoint inhibitors has been recognized as a separate entity of special consideration and management needs (74). Nonetheless, no late-onset or long-term safety concerns were reported with addition of PD-1 checkpoint inhibitors.

Our study is limited by several factors. First, a number of non-randomized clinical trials were included which may impart certain degree of inevitable heterogeneity. Second, certain studies involved small number of patients which contributed high heterogeneity to the pooled outcomes of ORR and adverse events of single-arm studies. Certain dissimilarities were noticed in these studies in reference to the type of agent (PD-1 or PD-L1 inhibitor), chemotherapy regimen, PD-L1 expression assessment assays, treatment combination (IT versus ICT) and line of therapy. Subgroup analysis were undertaken to address these issues; nonetheless, these differences may still impact pooled analysis.

## Conclusion

In summary, the results indicate that PD-L1 expression can successfully predict objective response rate and 2-year OS in mTNBC patients receiving PD-1/PD-L1 checkpoint inhibitors plus/minus chemotherapy. Short-term progression-free survival (1-year) is only predicted by PD-L1 expression when treatment is administered in the first-line setting and with chemotherapy. It can also predict the ORR, PFS and OS for PD-L1+ mTNBC patients receiving PD-1 checkpoint inhibitors plus chemotherapy as compared to chemotherapy alone. Overall, PD-L1 expression can be a useful biomarker of efficacy of PD-1/PD-L1 checkpoint inhibitors in mTNBC.

## Data availability statement

The original contributions presented in the study are included in the article/**Supplementary Material**. Further inquiries can be directed to the corresponding authors.

## Author contributions

MK, KD, MA, and BW designed the project, performed data extraction, and statistical analysis. MK wrote the initial manuscript. JL, AR, CC, ZH, WQ, YT, and YY provided critical assessment and supervision. All authors contributed to the article and approved the submitted version.
