# Supplementary material for: PD-L1 expression as biomarker of efficacy of PD-1/PD-L1 checkpoint inhibitors in metastatic triple negative breast cancer: A systematic review and meta-analysis
Source: Front Immunol. 2023 Mar 6;14:1060308. doi: 10.3389/fimmu.2023.1060308 (PMC10027008; doi:10.3389/fimmu.2023.1060308)
Supplement: Supplementary file 1 [file DataSheet_1.docx]

**Supplementary Appendix**

**PD-L1 expression as biomarker of efficacy of PD-1/PD-L1 checkpoint inhibitors in metastatic triple negative breast cancer: a systematic review and meta-analysis**

This appendix has been provided by the authors to give readers additional information about their work.

Table of Contents

[Supplementary Figure 1. Forest plot depicting objective response rate of metastatic triple-negative breast cancer treated with PD-1/PD-L1 checkpoint inhibition therapy. 2](#_Toc122278455)

[Supplementary Figure 2. Outcomes of efficacy of PD-1/PD-L1 checkpoint inhibition therapy in metastatic triple-negative breast cancer. 3](#_Toc122278456)

[Supplementary Figure 3. Forest plots depicting comparison of progression-free survival in PD-L1+ and PD-L1- metastatic triple-negative breast cancer between ICT and CT alone. 4](#_Toc122278457)

[Supplementary Figure 4. Subgroup analysis based on 1-year progression-free survival (PFS). 5](#_Toc122278458)

[Supplementary Figure 5. Subgroup analysis based on 2-year overall survival (OS). 6](#_Toc122278459)

[Supplementary Figure 6. Forest plot of any grade (A) and grade≥3 (B) treatment-related adverse events in metastatic triple-negative breast cancer treated with PD-1/PD-L1 checkpoint inhibition therapy. 7](#_Toc122278460)

[Supplementary Figure 7: Forest plot of comparison of adverse events in metastatic triple-negative breast cancer between patients receiving ICT and CT alone. 8](#_Toc122278461)

[Supplementary Figure 8. Forest plot of any grade (A) and grade≥3 (B) immune-related adverse events in metastatic triple-negative breast cancer treated with PD-1/PD-L1 checkpoint inhibition therapy. 9](#_Toc122278462)

[Supplementary Table 1. Methodological quality of included randomized controlled trials for prognosis based on Cochrane risk of bias tool. 10](#_Toc122278463)

[Supplementary Table 2. Methodological quality of included non-randomized studies for prognosis based on the methodological index for non-randomized studies (MINORS). 11](#_Toc122278464)

[Supplementary Table 3. Risk of bias assessment of included studies for prognostic analysis based on Quality In Prognosis Studies (QUIPS) tool. 12](#_Toc122278465)

[Supplementary Table 4. GRADE quality of evidence summary. 13](#_Toc122278466)

[Supplementary Table 5. PubMed search history. 14](#_Toc122278467)


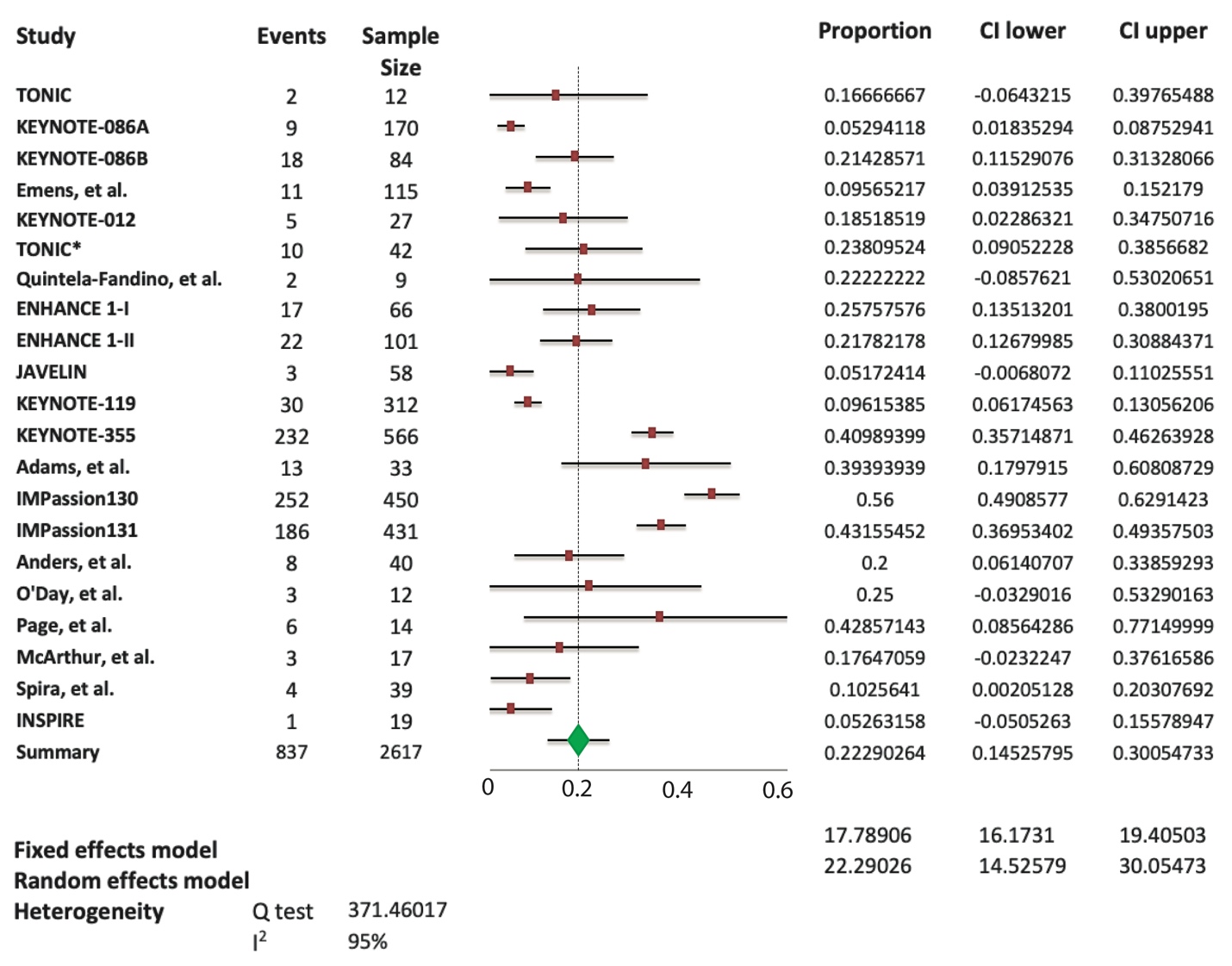


# **Supplementary Figure 1.** Forest plot depicting objective response rate of metastatic triple-negative breast cancer treated with PD-1/PD-L1 checkpoint inhibition therapy.

TONIC* indicates the participants with induction chemotherapy before immunotherapy.


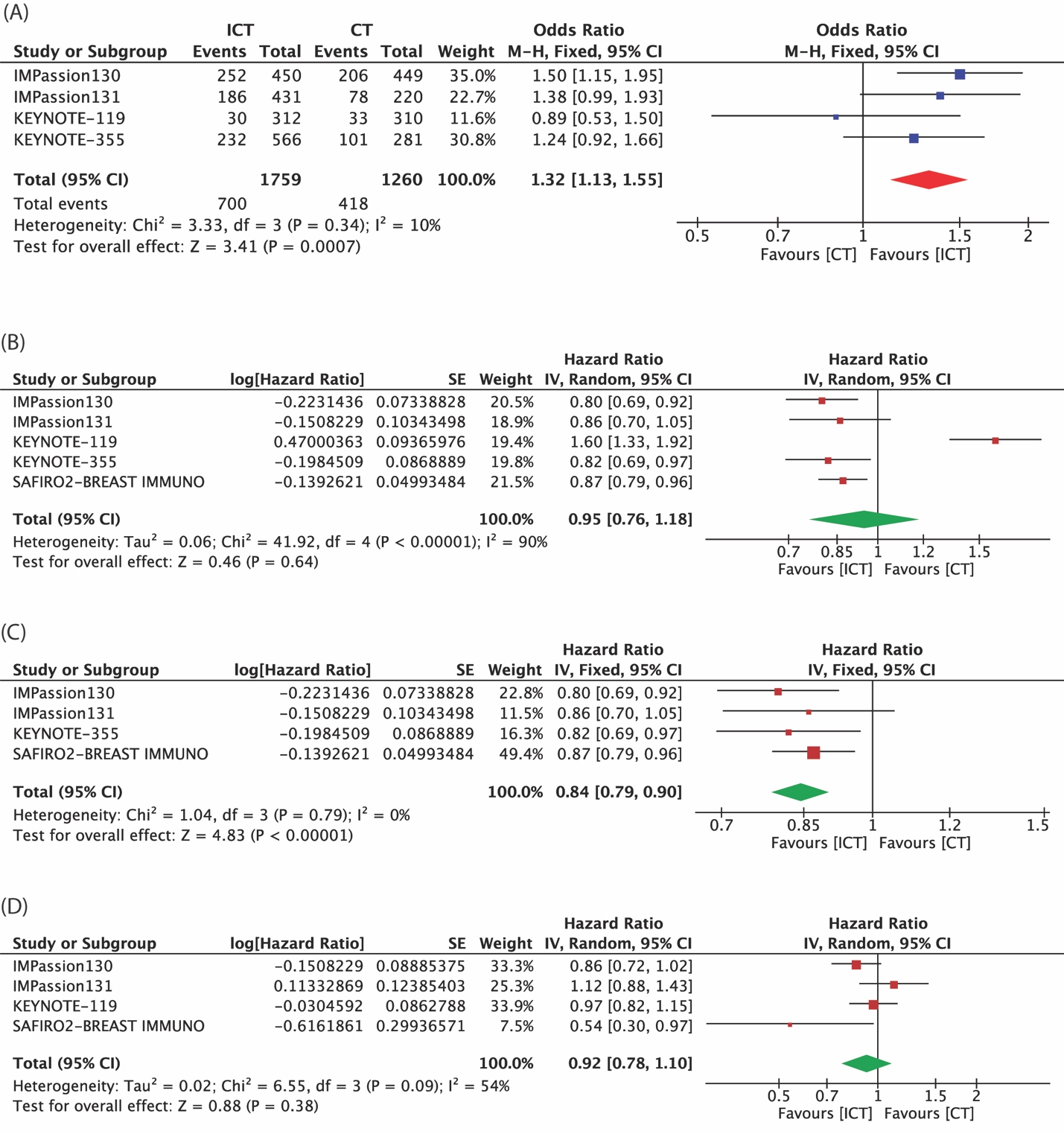


# **Supplementary Figure 2.** Outcomes of efficacy of PD-1/PD-L1 checkpoint inhibition therapy in metastatic triple-negative breast cancer.

**A)** Comparison of ICT versus CT according to objective response rate, **B)** Progression-free survival, **C)** Progression-free survival after exclusion of KEYNOTE-119 trial data, and **D)** Overall survival.

***Abbreviations:*** ICT = PD-1/PD-L1 checkpoint inhibitors plus chemotherapy; CT = chemotherapy alone

**
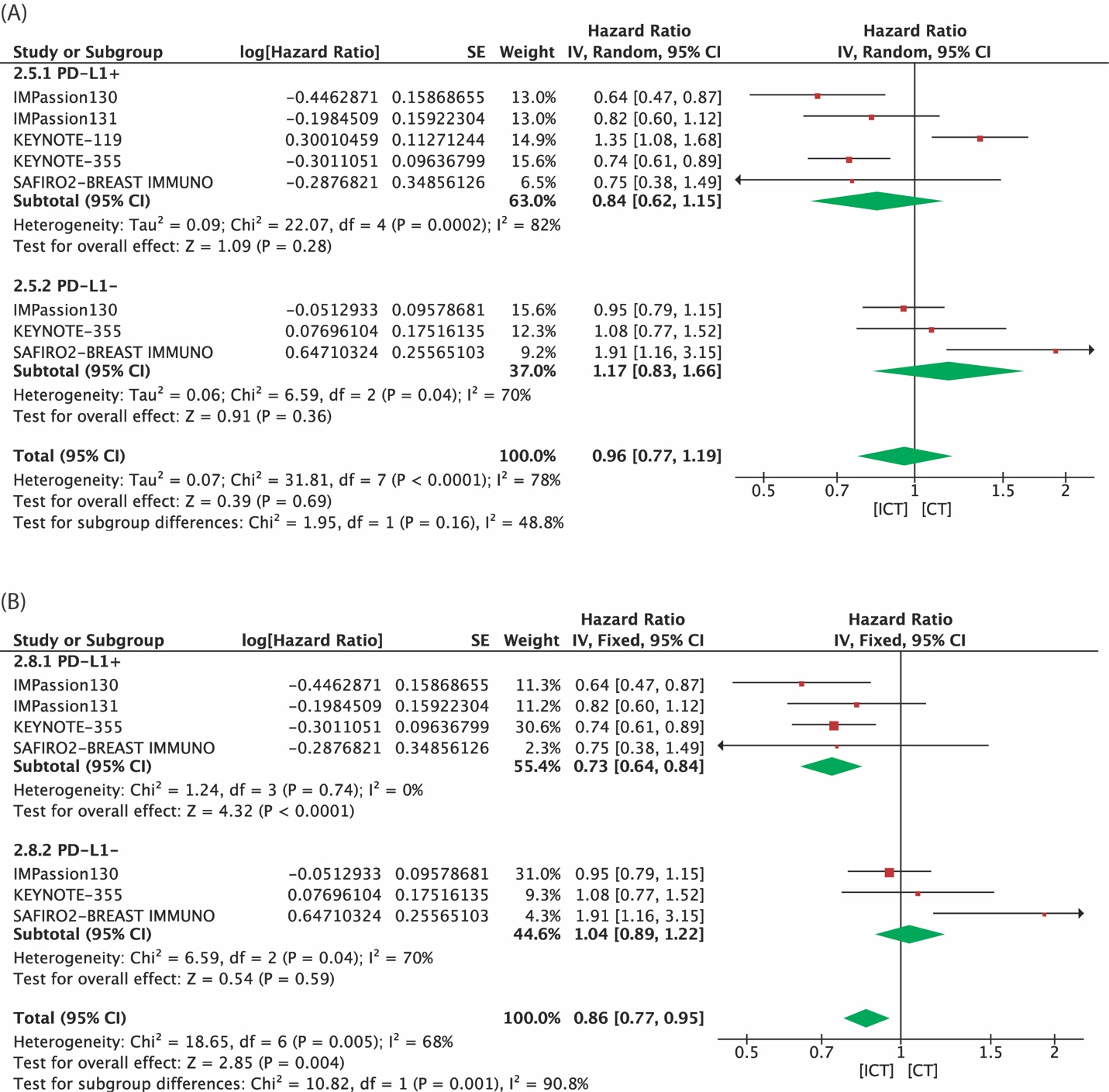
**

# **Supplementary Figure 3.** Forest plots depicting comparison of progression-free survival in PD-L1+ and PD-L1- metastatic triple-negative breast cancer between ICT and CT alone.

**A)** Comparison after application of random effects model, and **B)** after exclusion of KEYNOTE-119 trial.

***Abbreviations:*** ICT = PD-1/PD-L1 checkpoint inhibitors plus chemotherapy; CT = chemotherapy alone


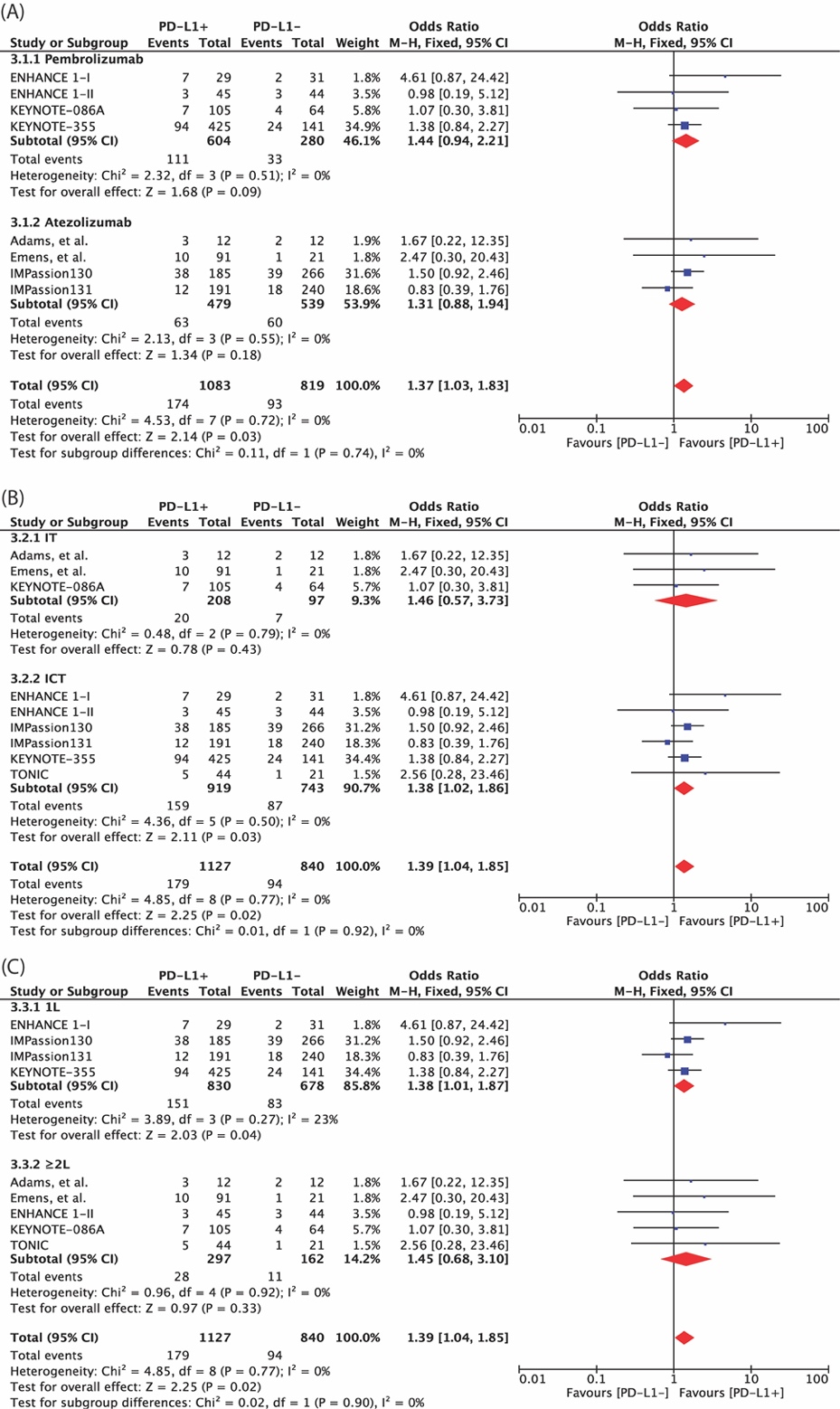


# **Supplementary Figure 4.** Subgroup analysis based on 1-year progression-free survival (PFS)**.**

**A)** Forest plot of comparison of 1-year PFS between PD-L1+ and PD-L1- metastatic triple-negative breast cancer treated with pembrolizumab and atezolizumab, B) IT alone and ICT, and C) treatment administered in first- and second-line setting.

***Abbreviations:*** ICT = PD-1/PD-L1 checkpoint inhibitors plus chemotherapy; IT = PD-1 checkpoint inhibitors

**
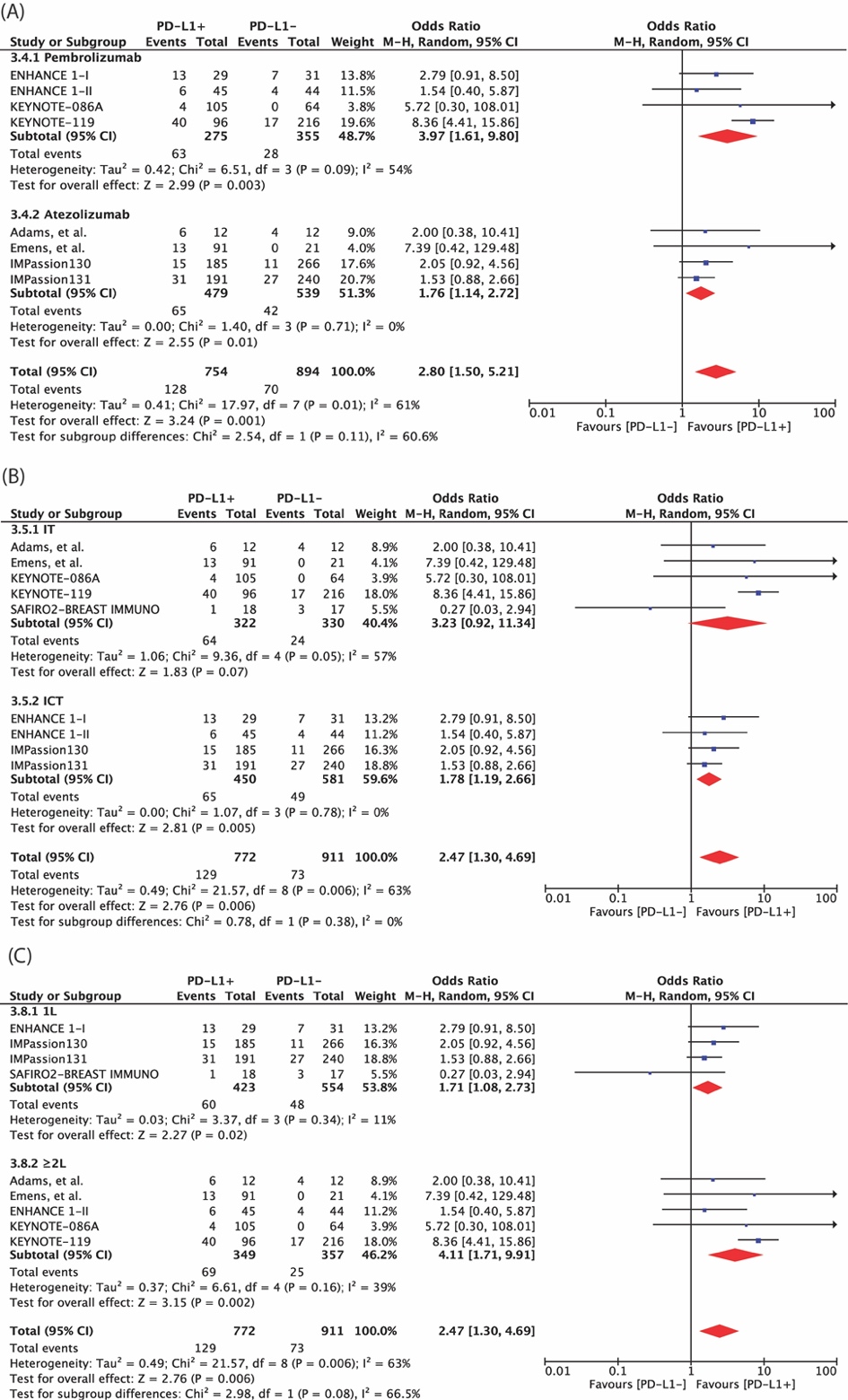
**

# **Supplementary Figure 5.** Subgroup analysis based on 2-year overall survival (OS)**.**

**A)** Forest plot of comparison of 1-year PFS between PD-L1+ and PD-L1- metastatic triple-negative breast cancer treated with pembrolizumab and atezolizumab, **B)** IT alone and ICT, and **C)** treatment administered in first- and second-line setting.

***Abbreviations:*** ICT = PD-1/PD-L1 checkpoint inhibitors plus chemotherapy; IT = PD-1 checkpoint inhibitors


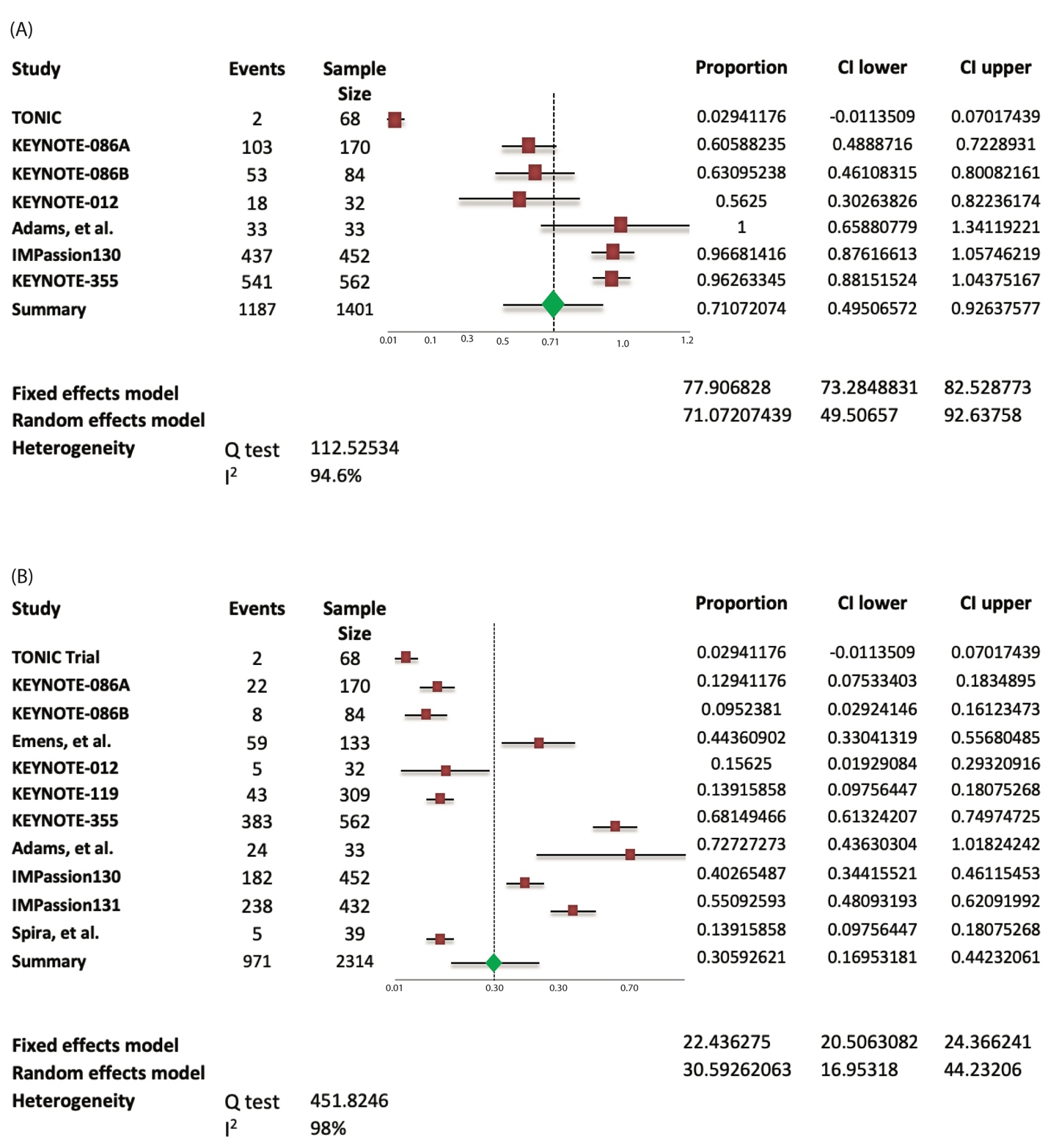


# **Supplementary Figure 6.** Forest plot of any grade (A) and grade≥3 (B) treatment-related adverse events in metastatic triple-negative breast cancer treated with PD-1/PD-L1 checkpoint inhibition therapy.


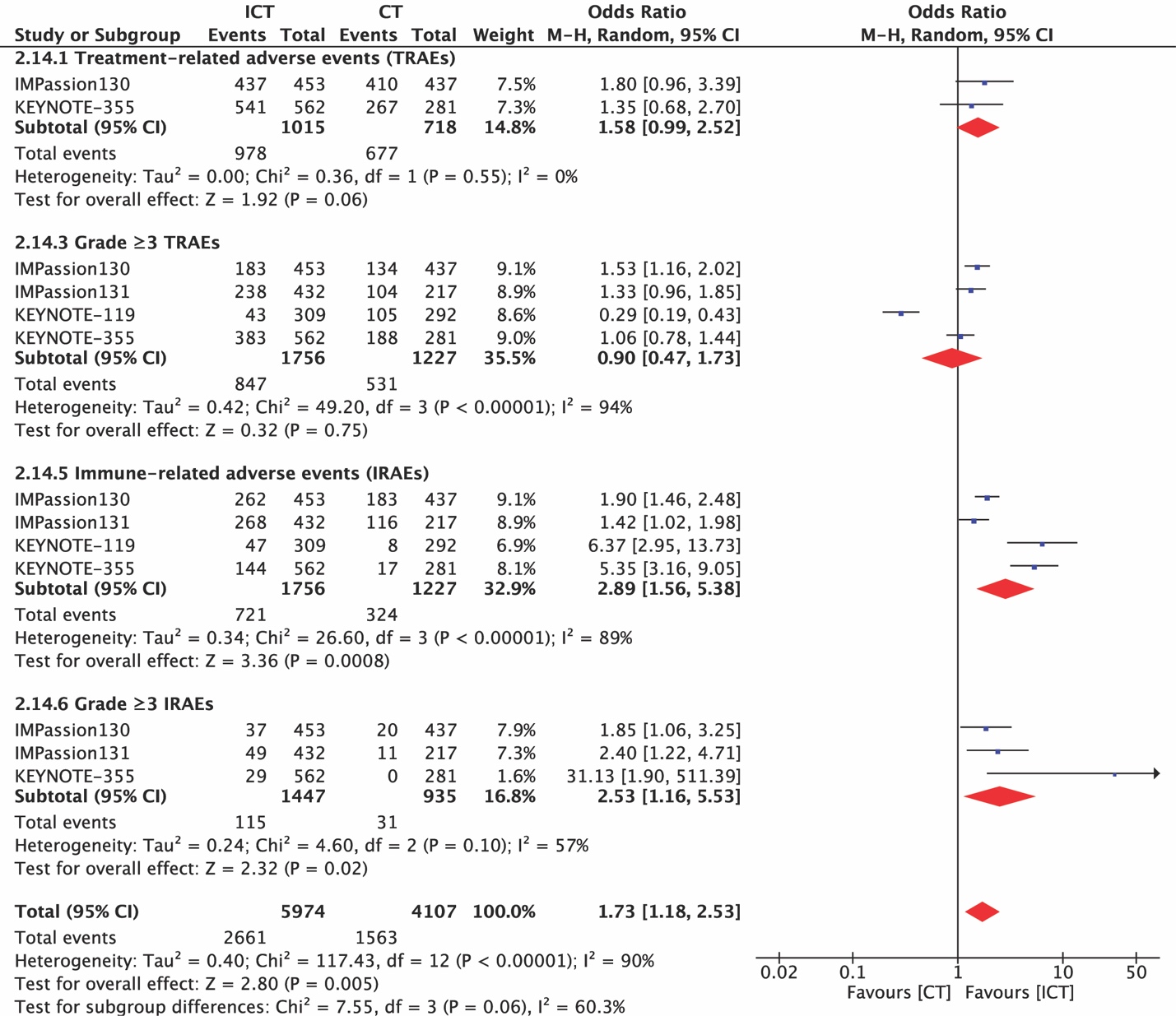


# **Supplementary Figure 7**: Forest plot of comparison of adverse events in metastatic triple-negative breast cancer between patients receiving ICT and CT alone.

***Abbreviations:*** ICT = PD-1 /PD-L1 checkpoint inhibitors plus chemotherapy; CT = chemotherapy alone


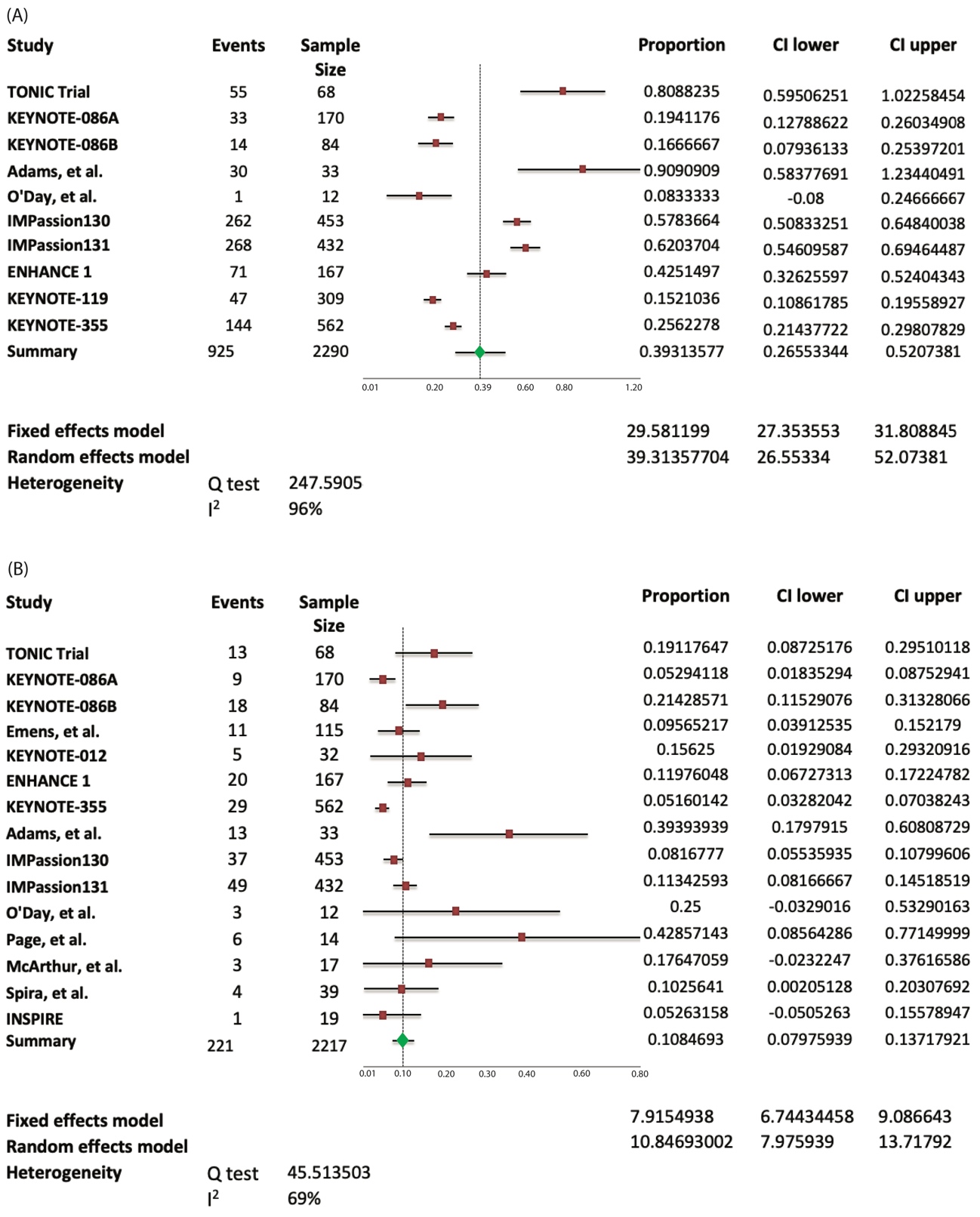


# **Supplementary Figure 8.** Forest plot of any grade (A) and grade≥3 (B) immune-related adverse events in metastatic triple-negative breast cancer treated with PD-1/PD-L1 checkpoint inhibition therapy.

# **Supplementary Table 1.** Methodological quality of included randomized controlled trials for prognosis based on Cochrane risk of bias tool.


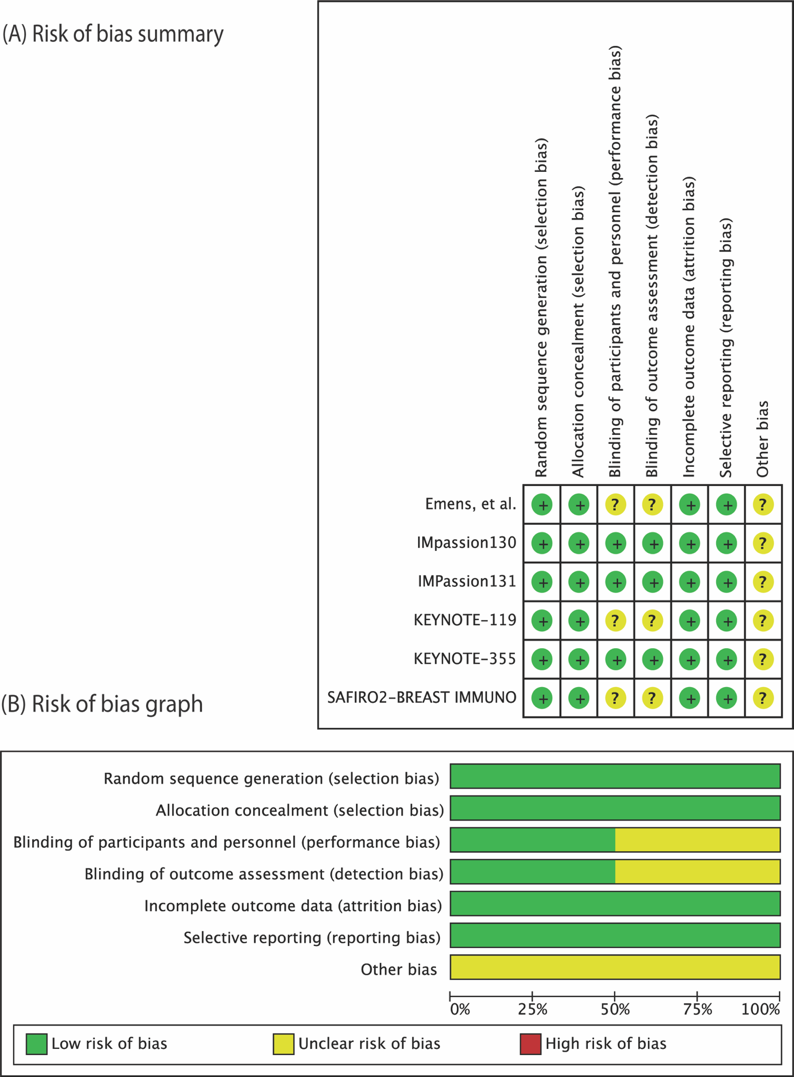


**Supplementary Table 2.** Methodological quality of included non-randomized studies for prognosis based on the methodological index for non-randomized studies (MINORS).

| Study | A clearly stated aim | Inclusion of consecutive patients | Prospective collection of data | Endpoints appropriate to the aim of the study | Unbiased assessment of the study endpoint | Follow-up period appropriate to the aim of the study | Loss to follow up less than 5% | Prospective calculation of the study size | Total Score^†^ |
| --- | --- | --- | --- | --- | --- | --- | --- | --- | --- |
| Adams, et al. | 2 | 2 | 2 | 2 | 1 | 2 | 2 | 2 | 15 |
| Anders, et al. | 2 | 2 | 2 | 1 | 1 | 0 | 0 | 0 | 9 |
| O'Day, et al. | 2 | 2 | 2 | 1 | 1 | 0 | 0 | 0 | 9 |
| Page, et al. | 2 | 2 | 2 | 1 | 1 | 0 | 2 | 0 | 11 |
| McArthur, et al. | 2 | 2 | 2 | 1 | 1 | 0 | 2 | 0 | 11 |
| Spira, et al. | 2 | 2 | 2 | 2 | 1 | 0 | 0 | 0 | 9 |
| INSPIRE | 2 | 2 | 2 | 2 | 1 | 1 | 2 | 0 | 12 |
| KEYNOTE-086A | 2 | 2 | 2 | 2 | 1 | 1 | 1 | 2 | 13 |
| KEYNOTE-086B | 2 | 2 | 2 | 2 | 1 | 2 | 2 | 2 | 15 |
| JAVELIN | 2 | 2 | 2 | 2 | 1 | 1 | 1 | 2 | 13 |
| Quintela-Fandino, et al. | 2 | 2 | 2 | 2 | 1 | 0 | 2 | 0 | 11 |
| ENHANCE 1 | 2 | 2 | 2 | 2 | 1 | 0 | 1 | 0 | 10 |
| TONIC | 2 | 2 | 2 | 2 | 1 | 2 | 1 | 2 | 14 |
| KEYNOTE-012 | 2 | 2 | 2 | 2 | 1 | 1 | 1 | 2 | 13 |

† The items are scored 0 (not reported), 1 (reported but inadequate) or 2 (reported and adequate). The global ideal score being 16 for non-comparative studies and 24 for comparative studies.

# **Supplementary Table 3.** Risk of bias assessment of included studies for prognostic analysis based on Quality In Prognosis Studies (QUIPS) tool.

H: High risk of bias; M: Moderate risk of bias; L: Low risk of bias.

| Study | Study participation | Study Attrition | Prognostic Factor | Outcome Measurement | Study Confounding | Statistical Analysis and Reporting |
| --- | --- | --- | --- | --- | --- | --- |
| TONIC | M | L | L | L | M | L |
| KEYNOTE-086A | L | L | L | L | M | L |
| KEYNOTE-086B | L | L | L | L | M | L |
| Adams, et al. | M | L | L | L | M | L |
| JAVELIN | L | L | L | L | H | L |
| ENHANCE 1 | H | H | H | M | M | M |
| Emens, et al. | M | L | H | M | M | M |
| IMPassion130 | L | L | L | L | M | L |
| IMPassion131 | L | L | L | L | M | L |
| KEYNOTE-119 | L | L | L | L | H | L |
| KEYNOTE-355 | L | L | L | M | M | L |
| SAFIR02-BREAST IMMUNO | M | L | L | M | M | L |

# **Supplementary Table 4.** GRADE quality of evidence summary.

| **Quality assessment** | | | | | | | **No. of patients** | **Effect**  **OR (95% CI)** | **Certainty** | **Importance** |
| --- | --- | --- | --- | --- | --- | --- | --- | --- | --- | --- |
| **No. of studies** | **Study design** | **Risk of bias** | **Inconsistency** | **Imprecision** | **Indirectness** | **Publication bias** |  |  |  |  |
| **PD-L1+ vs PD-L1- patients – ORR** | | | | | | | | | | |
| 9 | 5 randomised, 4 non-randomized trials | serious^a^ | - | - | - | - | 2,077  (1130/947) | 1.78 (1.45-2.19) | ◯⨁⨁⨁⨁ Moderate | CRITICAL |
| **PD-L1+ vs PD-L1- patients – 1-year PFS** | | | | | | | | | | |
| 8 | 4 randomised, 4 non-randomized trials | serious^a^ | - | - | - | - | 1,967  (1127/840) | **1.39** (1.04-1.85) | ◯⨁⨁⨁⨁ Moderate | CRITICAL |
| **PD-L1+ vs PD-L1- patients – 2-year OS** | | | | | | | | | | |
| 8 | 5 randomised, 3 non-randomized trials | serious^a^ | serious^b^ | - | - | - | 1,683  (772/911) | **2.47**  (1.30-4.69) | ◯◯⨁⨁⨁ Low | CRITICAL |
|  | | | | | | | | | | |

**a difference in investigating agents, PD-L1 assay, first-line/second-line treatment induction, and addition of chemotherapy.**

**b Inconsistency index *I*^2^ >50% for the effect.
c Wide 95% CI of the effect.
d Funnel Plot indicates publication bias.**

# **Supplementary Table 5.** PubMed search history.

| Search number | Query | Sort By | Filters | Search Details | Results | Time |
| --- | --- | --- | --- | --- | --- | --- |
| 3 | (((((((((((((((((((((((((((((((breast cancer[MeSH Terms])) OR (triple negative breast cancer[Title/Abstract])) OR (TNBC[Title/Abstract])) OR (basal like breast cancer[Title/Abstract])) OR (breast neoplasms[Title/Abstract])) OR (metastatic breast cancer[Title/Abstract])) OR (advanced breast cancer[Title/Abstract])) OR (metastatic tnbc[Title/Abstract])) OR (invasive breast cancer[Title/Abstract])) OR (breast carcinoma[Title/Abstract])) AND (Immunotherapy[Title/Abstract])) OR (immune checkpoint inhibition[Title/Abstract])) OR (ici therapy[Title/Abstract])) OR (pd 1 inhibitor[Title/Abstract])) OR (nivolumab[Title/Abstract])) OR (Opdivo[Title/Abstract])) OR (pembrolizumab[Title/Abstract])) OR (Keytruda[Title/Abstract])) OR (Atezolizumab[Title/Abstract])) OR (Tecentriq[Title/Abstract])) OR (Avelumab[Title/Abstract])) OR (MSB0010718C[Title/Abstract])) OR (Durvalumab[Title/Abstract])) OR (MEDI4736[Title/Abstract])) OR (bms 936559[Title/Abstract])) OR (pd l1 inhibitor[Title/Abstract])) OR (anti pd 1 therapy[Title/Abstract])) OR (anti pd l1 therapy[Title/Abstract]))) OR (programmed cell death 1 receptor[MeSH Terms])) OR (b7 h1 antigen[MeSH Terms]) |  | Clinical Trial, Randomized Controlled Trial | ((("breast neoplasms"[MeSH Terms] OR "triple negative breast cancer"[Title/Abstract] OR "TNBC"[Title/Abstract] OR "basal like breast cancer"[Title/Abstract] OR "breast neoplasms"[Title/Abstract] OR "metastatic breast cancer"[Title/Abstract] OR "advanced breast cancer"[Title/Abstract] OR "metastatic tnbc"[Title/Abstract] OR "invasive breast cancer"[Title/Abstract] OR "breast carcinoma"[Title/Abstract]) AND "Immunotherapy"[Title/Abstract]) OR "immune checkpoint inhibition"[Title/Abstract] OR "ici therapy"[Title/Abstract] OR "pd 1 inhibitor"[Title/Abstract] OR "nivolumab"[Title/Abstract] OR "Opdivo"[Title/Abstract] OR "pembrolizumab"[Title/Abstract] OR "Keytruda"[Title/Abstract] OR "Atezolizumab"[Title/Abstract] OR "Tecentriq"[Title/Abstract] OR "Avelumab"[Title/Abstract] OR "MSB0010718C"[Title/Abstract] OR "Durvalumab"[Title/Abstract] OR "MEDI4736"[Title/Abstract] OR "bms 936559"[Title/Abstract] OR "pd l1 inhibitor"[Title/Abstract] OR "anti pd 1 therapy"[Title/Abstract] OR "anti pd l1 therapy"[Title/Abstract] OR "programmed cell death 1 receptor"[MeSH Terms] OR "b7 h1 antigen"[MeSH Terms]) AND (clinicaltrial[Filter] OR randomizedcontrolledtrial[Filter]) | 1,806 | 0:22:29 |
| 2 | (((((((((((((((((((((((((((((((breast cancer[MeSH Terms])) OR (triple negative breast cancer[Title/Abstract])) OR (TNBC[Title/Abstract])) OR (basal like breast cancer[Title/Abstract])) OR (breast neoplasms[Title/Abstract])) OR (metastatic breast cancer[Title/Abstract])) OR (advanced breast cancer[Title/Abstract])) OR (metastatic tnbc[Title/Abstract])) OR (invasive breast cancer[Title/Abstract])) OR (breast carcinoma[Title/Abstract])) AND (Immunotherapy[Title/Abstract])) OR (immune checkpoint inhibition[Title/Abstract])) OR (ici therapy[Title/Abstract])) OR (pd 1 inhibitor[Title/Abstract])) OR (nivolumab[Title/Abstract])) OR (Opdivo[Title/Abstract])) OR (pembrolizumab[Title/Abstract])) OR (Keytruda[Title/Abstract])) OR (Atezolizumab[Title/Abstract])) OR (Tecentriq[Title/Abstract])) OR (Avelumab[Title/Abstract])) OR (MSB0010718C[Title/Abstract])) OR (Durvalumab[Title/Abstract])) OR (MEDI4736[Title/Abstract])) OR (bms 936559[Title/Abstract])) OR (pd l1 inhibitor[Title/Abstract])) OR (anti pd 1 therapy[Title/Abstract])) OR (anti pd l1 therapy[Title/Abstract]))) OR (programmed cell death 1 receptor[MeSH Terms])) OR (b7 h1 antigen[MeSH Terms]) |  | Clinical Trial | ((("breast neoplasms"[MeSH Terms] OR "triple negative breast cancer"[Title/Abstract] OR "TNBC"[Title/Abstract] OR "basal like breast cancer"[Title/Abstract] OR "breast neoplasms"[Title/Abstract] OR "metastatic breast cancer"[Title/Abstract] OR "advanced breast cancer"[Title/Abstract] OR "metastatic tnbc"[Title/Abstract] OR "invasive breast cancer"[Title/Abstract] OR "breast carcinoma"[Title/Abstract]) AND "Immunotherapy"[Title/Abstract]) OR "immune checkpoint inhibition"[Title/Abstract] OR "ici therapy"[Title/Abstract] OR "pd 1 inhibitor"[Title/Abstract] OR "nivolumab"[Title/Abstract] OR "Opdivo"[Title/Abstract] OR "pembrolizumab"[Title/Abstract] OR "Keytruda"[Title/Abstract] OR "Atezolizumab"[Title/Abstract] OR "Tecentriq"[Title/Abstract] OR "Avelumab"[Title/Abstract] OR "MSB0010718C"[Title/Abstract] OR "Durvalumab"[Title/Abstract] OR "MEDI4736"[Title/Abstract] OR "bms 936559"[Title/Abstract] OR "pd l1 inhibitor"[Title/Abstract] OR "anti pd 1 therapy"[Title/Abstract] OR "anti pd l1 therapy"[Title/Abstract] OR "programmed cell death 1 receptor"[MeSH Terms] OR "b7 h1 antigen"[MeSH Terms]) AND (clinicaltrial[Filter]) | 1,806 | 0:22:22 |
| 1 | (((((((((((((((((((((((((((((((breast cancer[MeSH Terms])) OR (triple negative breast cancer[Title/Abstract])) OR (TNBC[Title/Abstract])) OR (basal like breast cancer[Title/Abstract])) OR (breast neoplasms[Title/Abstract])) OR (metastatic breast cancer[Title/Abstract])) OR (advanced breast cancer[Title/Abstract])) OR (metastatic tnbc[Title/Abstract])) OR (invasive breast cancer[Title/Abstract])) OR (breast carcinoma[Title/Abstract])) AND (Immunotherapy[Title/Abstract])) OR (immune checkpoint inhibition[Title/Abstract])) OR (ici therapy[Title/Abstract])) OR (pd 1 inhibitor[Title/Abstract])) OR (nivolumab[Title/Abstract])) OR (Opdivo[Title/Abstract])) OR (pembrolizumab[Title/Abstract])) OR (Keytruda[Title/Abstract])) OR (Atezolizumab[Title/Abstract])) OR (Tecentriq[Title/Abstract])) OR (Avelumab[Title/Abstract])) OR (MSB0010718C[Title/Abstract])) OR (Durvalumab[Title/Abstract])) OR (MEDI4736[Title/Abstract])) OR (bms 936559[Title/Abstract])) OR (pd l1 inhibitor[Title/Abstract])) OR (anti pd 1 therapy[Title/Abstract])) OR (anti pd l1 therapy[Title/Abstract]))) OR (programmed cell death 1 receptor[MeSH Terms])) OR (b7 h1 antigen[MeSH Terms]) |  |  | (("breast neoplasms"[MeSH Terms] OR "triple negative breast cancer"[Title/Abstract] OR "TNBC"[Title/Abstract] OR "basal like breast cancer"[Title/Abstract] OR "breast neoplasms"[Title/Abstract] OR "metastatic breast cancer"[Title/Abstract] OR "advanced breast cancer"[Title/Abstract] OR "metastatic tnbc"[Title/Abstract] OR "invasive breast cancer"[Title/Abstract] OR "breast carcinoma"[Title/Abstract]) AND "Immunotherapy"[Title/Abstract]) OR "immune checkpoint inhibition"[Title/Abstract] OR "ici therapy"[Title/Abstract] OR "pd 1 inhibitor"[Title/Abstract] OR "nivolumab"[Title/Abstract] OR "Opdivo"[Title/Abstract] OR "pembrolizumab"[Title/Abstract] OR "Keytruda"[Title/Abstract] OR "Atezolizumab"[Title/Abstract] OR "Tecentriq"[Title/Abstract] OR "Avelumab"[Title/Abstract] OR "MSB0010718C"[Title/Abstract] OR "Durvalumab"[Title/Abstract] OR "MEDI4736"[Title/Abstract] OR "bms 936559"[Title/Abstract] OR "pd l1 inhibitor"[Title/Abstract] OR "anti pd 1 therapy"[Title/Abstract] OR "anti pd l1 therapy"[Title/Abstract] OR "programmed cell death 1 receptor"[MeSH Terms] OR "b7 h1 antigen"[MeSH Terms] | 31,350 | 0:22:14 |
